# Supplementary material for: Non-Planar Geometrical Effects on the Magnetoelectrical Signal in a Three-Dimensional Nanomagnetic Circuit
Source: ACS Nano. 2021 Apr 13;15(4):6765–73. doi: 10.1021/acsnano.0c10272 (PMC8155340; doi:10.1021/acsnano.0c10272)
Supplement: Supplementary file 1 — nn0c10272_si_001.pdf [file nn0c10272_si_001.pdf]

Supporting Information

Non-Planar Geometrical Effects on the Magnetoelectrical Signal in a Three-Dimensional Nanomagnetic Circuit

Fanfan Meng,[†] *Claire Donnelly,*[*,†] *Claas Abert,*[‡,§] *Luka Skoric,*[†] *Stuart Holmes,*[||] *Zhuocong Xiao,*[^] *Jung-Wei Liao,*[†] *Peter J. Newton,*[†] *Crispin H.W. Barnes,*[†] *Dédalo Sanz-Hernández,*[†,#] *Aurelio Hierro-Rodriguez,*[††,‡‡] *Dieter Suess,*[‡,§] *Russell P. Cowburn,*[†] *and Amalio Fernández-Pacheco,*[**,†,‡‡]

[†]Cavendish Laboratory, University of Cambridge, Cambridge, CB3 0HE, UK

[‡]Faculty of Physics, University of Vienna, Vienna, 1090, Austria

[§]Research Platform MMM Mathematics-Magnetism-Materials, University of Vienna, Vienna, 1090, Austria

[||]London Centre for Nanotechnology, UCL, London, WC1H 0AH, UK

[^]Nanoscience Centre, University of Cambridge, Cambridge, CB3 0FF, UK

[#]Unité Mixte de Physique, CNRS, Thales, Université Paris-Saclay, Palaiseau, 91767, France

[††]Depto. Física, Universidad de Oviedo, Oviedo, 33007, Spain

[‡‡]SUPA, School of Physics and Astronomy, University of Glasgow, Glasgow, G12 8QQ, UK

* cd691@cam.ac.uk
** amalio.fernandez-pacheco@glasgow.ac.uk

In order to comply with EPSRC policy, the metadata associated to this publication can be found here: https://doi.org/10.17863/CAM.65676

S1 Geometrical details of the nanobridge inferred from scanning electron microscopy (SEM) images

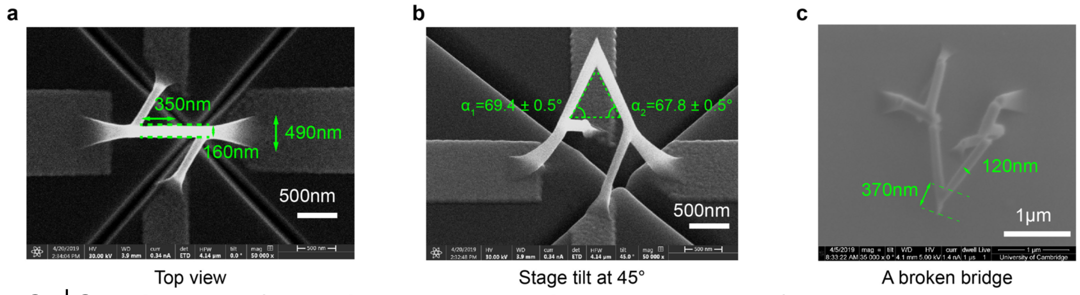

Figure S1 | SEM images of the printed 3D nanobridge. a, Top view of the measured bridge. **b**, View of the measured bridge with stage tilt of 45°. **c**, Top view of a broken bridge fabricated under the same conditions.

The geometrical information of the bridge is obtained from SEM images taken from different angles. This information is used to create the 3D CAD-based model and FEM mesh used in the magnetoresistance calculation. From the top view image (**Figure S1a**), the width of the main bridge is approximately 160 nm and the width of the 'enlarged base' is approximately 490 nm. The projection of the probe region on the substrate plane is approximately $350 \times 2 = 700$ nm. By tilting the sample stage by 45° (**Figure S1b**), we can calculate the angle, α, between the bridge and substrate. We have measured the projected angle α' to be 62° ± 0.5° and 60° ± 0.5° from the SEM image, which are the 45° projection to the substrate plane. Hence, the real angle can be calculated as $\alpha = \tan^{-1}\left[\frac{\tan(\alpha\prime)}{\cos(45°)}\right]$ to be 69.4 ± 0.56° and 67.8 ± 0.56°. From this analysis we determine that there is a 1.6 ± 0.8° difference in the angle formed by the legs and the x-axis. From the top view perspective in **Figure S1a**, we further estimate the symmetry of the structure by considering the boundary lines of the probed region (green dash lines) which are parallel to each other, indicating that any deviation of the structure from the x-axis is below the SEM resolution. As a result, we conclude that the degree of asymmetry is very small in our structure. With the known α, the length of one side of the probed region is estimated to be 1023 nm. Finally, the thickness of the measured bridge is estimated from another broken bridge (**Figure S1c**) that is grown under the same condition, and where the thickness is about 120 nm for the main bridge and 370 nm for the apex region.

S2 Macrospin model

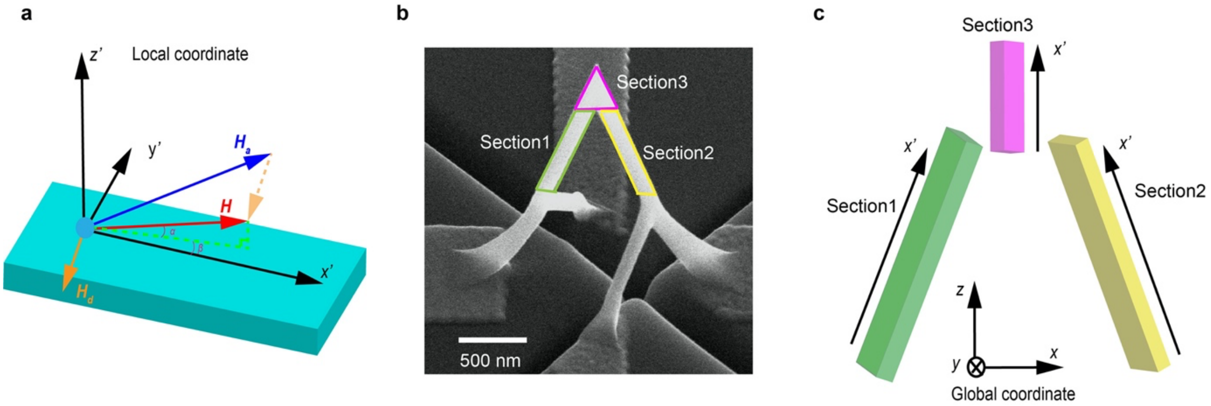

Figure S2 | The macrospin model. a, The applied field $\boldsymbol{H_a}$, the demagnetising field $\boldsymbol{H_d}$ and the effective field $\boldsymbol{H}$ are considered for a nanowire in the local coordinate system. **b-c,** Three nanowires forming the nanobridge, as considered in the model.

S2.1 Determination of *M* in a single domain nanowire at high fields

To calculate the magnetoresistance of the 3D nanobridge, we first need to determine its magnetisation. A macrospin model is used here to determine the magnetisation in the nanobridge at high fields (± 4 T) in a simple, computationally efficient way. Before studying the 3D nanobridge, we first consider how to determine the magnetisation in a single domain nanowire with a known applied field by minimising the sum of Zeeman and demagnetising energies.

$$E = -\mu_0 \boldsymbol{H_a} \cdot \boldsymbol{M} - \frac{1}{2}\mu_0 \boldsymbol{H_d} \cdot \boldsymbol{M}$$

As shown in **Figure S2a**, the magnetisation $\boldsymbol{M}$ can be written within the local coordinate system *x'y'z'* as,

$$\boldsymbol{M} = M_s \begin{pmatrix} \cos(\alpha)\cos(\beta) \\ \cos(\alpha)\sin(\beta) \\ \sin(\alpha) \end{pmatrix}$$

where α is the angle between $\boldsymbol{M}$ and the *x'y'*-plane, β is the angle between the projection of $\boldsymbol{M}$ on the *x'y'*-plane with the *x'*-axis, and M_s is the saturation magnetisation of the material. The demagnetising field is the magnetic field generated by the magnetisation within the ferromagnet material and is determined uniquely by the geometrical shape of the body as[1]

$$\boldsymbol{H_d} = -N\boldsymbol{M}$$

The demagnetising field lies along the opposite direction of $\vec{M}$, and has a magnitude proportional to $\boldsymbol{M}$. The coefficient N is the demagnetising factor and has three components [$N_{x'}$ $N_{y'}$ $N_{z'}$], which are associated with the three-principal axes of the geometry, and they obey the general constraint $N_{x'} + N_{y'} + N_{z'} = 1$. So, the demagnetising field can be written as

$$\boldsymbol{H_d} = -M_s \begin{pmatrix} N_{x'} \cos(\alpha)\cos(\beta) \\ N_{y'} \cos(\alpha)\sin(\beta) \\ N_{z'} \sin(\alpha) \end{pmatrix}$$

The applied field can be written as

$$\boldsymbol{H_a} = H_a \begin{pmatrix} a_{x'} \\ a_{y'} \\ a_{z'} \end{pmatrix}$$

where $[a_{x'}\ a_{y'}\ a_{z'}]$ is the known unit vector for the applied field.

The total energy E can be written as:

$$E = -\mu_0 H_a M_s [a_{x'} \cos(\alpha)\cos(\beta) + a_{y'} \cos(\alpha)\sin(\beta) + a_{z'} \sin(\alpha)] + \frac{1}{2}\mu_0 H_a {M_s}^2 [N_{x'} \cos^2(\alpha)\cos^2(\beta) + N_{y'} \cos^2(\alpha)\sin^2(\beta) + N_{z'} \sin^2(\beta)]$$

By solving the two partial differential equations below, we can determine α, β and hence the direction of the magnetisation vector $\boldsymbol{M}$.

$$\frac{\partial E}{\partial \alpha} = -H_a[-a_{x'} \cos(\beta)\sin(\alpha) - a_{y'} \sin(\beta)\sin(\alpha) + a_{z'} \cos(\alpha)] + M_s \sin(\alpha)\cos(\alpha) \left[-N_{x'} \cos^2(\beta) - N_{y'} \sin^2(\beta) + N_{z'}\right] = 0$$

$$\frac{\partial E}{\partial \beta} = -H_a\left[-a_{x'} \cos(\alpha)\sin(\beta) + a_{y'} \cos(\alpha)\cos(\beta)\right] + M_s \cos^2(\alpha)\sin(\beta)\cos(\beta) \left[-N_{x'} + N_{y'}\right] = 0$$

S2.2 Determination of the demagnetising factor for each section of the bridge

To apply the macrospin model of a single nanowire to the nanobridge, we assume the bridge is made up of three single-domain sections as shown in **Figure S2 b, c**. To do this, we need to determine the demagnetising factor for each section. To get a reasonable estimation of demagnetising factor for each section, we have simulated the demagnetising field ($\boldsymbol{H_d}$) for fields applied from $\theta = 0°$ to $\theta = 90°$ using a finite element method based on the magnum.fe package.[2] The saturation magnetisation, M_s, used in this simulation is 1.67 T,[3] and the results are shown in **Figure S3**. The grey arrows indicate the direction of $\boldsymbol{H_d}$ and the colour scale represents the *x*-component of $\boldsymbol{H_d}$. We also plot the average magnitude of $\boldsymbol{H_d}$ in each section of the bridge in **Figure S4**.

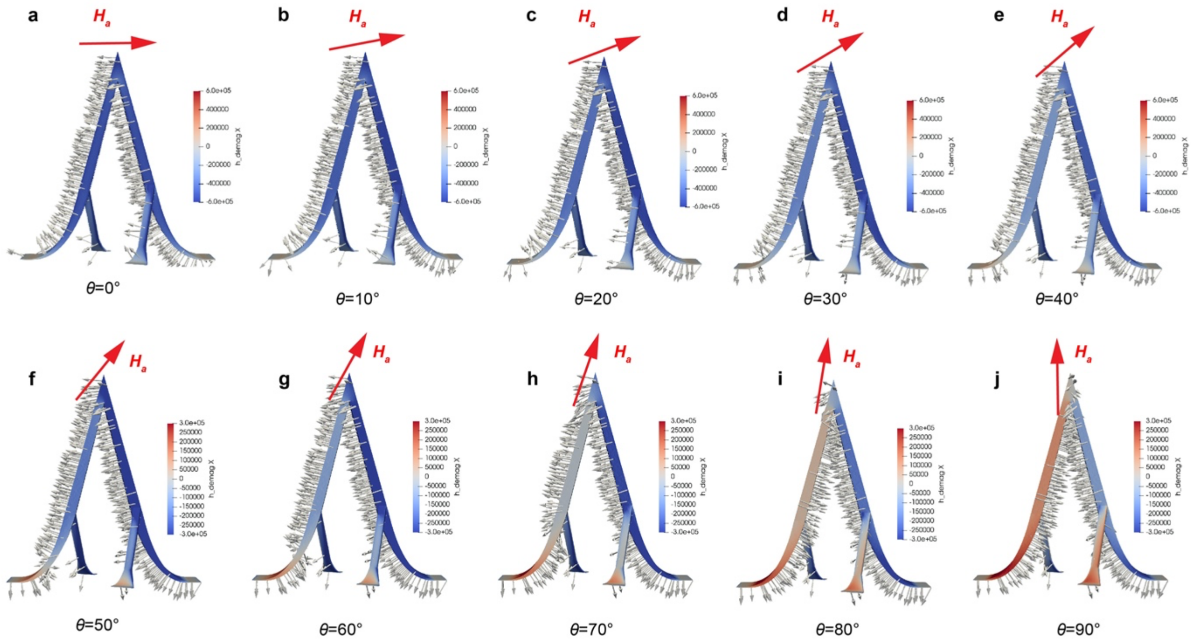

Figure S3 | Magnum.fe simulations of the demagnetising field H_d for H_a applied in different directions. The grey arrows indicate the direction of H_d (note that the lengths of the arrows do not represent the magnitude of the demagnetising field) and the blue-red colour scale indicates the *x*-component of the demagnetising field.

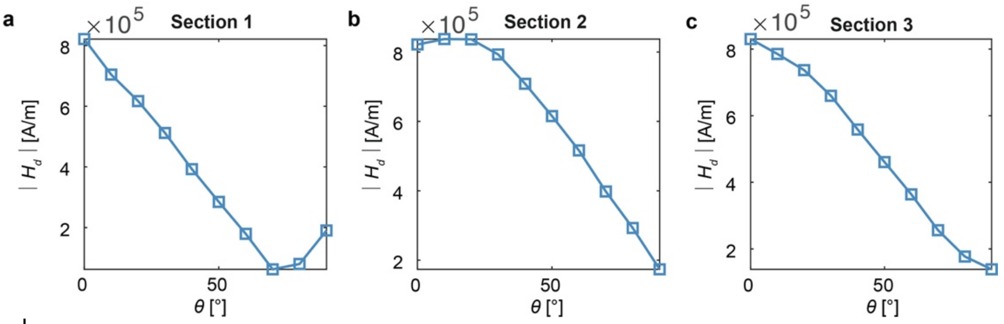

Figure S4 | The averaged magnitude of demagnetising field, H_d, from the simulation for the three sections marked in Figure S2b. a, Section 1. **b,** Section 2. **c,** Section 3.

For Section 1 (**Figure S4a**), there is a minimum in $|\boldsymbol{H_d}|$ at $\theta = 70°$. As shown in **Figure S3h**, when the field is applied at $\theta = 70°$, $\boldsymbol{H_a}$ is parallel to the long axis (easy) of the section 1, and hence gives the smallest $\boldsymbol{H_d}$. On the contrary, for Section 2, $|\boldsymbol{H_d}|$ peaks at $\theta = 20°$ (**Figure S4b**), where, as shown in **Figure S3c**, $\boldsymbol{H_a}$ is parallel to the hard axis of the Section 2, leading to a maximum in $\boldsymbol{H_d}$. Due to the unusual geometry of section 3, it is not immediately clear which is the easy or hard axis. However, from **Figure S4c**, we see $|\boldsymbol{H_d}|$ decreases with increasing θ, which indicates that its easy axis is aligned with the $\theta = 90°$ direction.

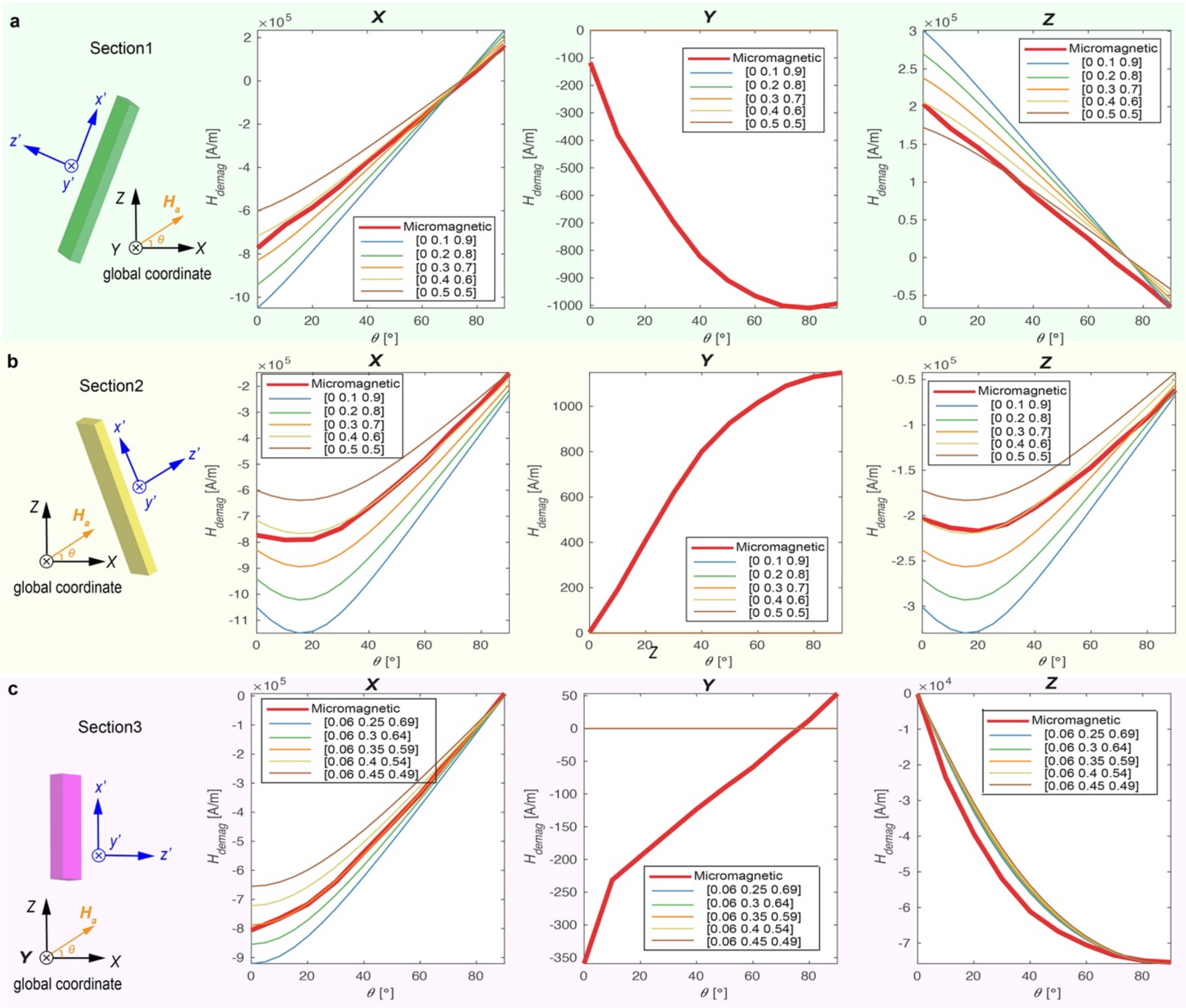

Figure S5 | **Comparison of the averaged *x*, *y*, *z* components of the demagnetising field, $\mathbf{H_d}$, obtained from macrospin and micromagnetic simulations a,** Section 1. **b,** Section 2. **c,** Section 3. For each section, the demagnetising field of micromagnetic simulations is given in red and used to identify the optimal demagnetising factor for the structure.

To determine the demagnetising factor for each section, we plot the x, y, z components (in the global coordinate system) of the simulated $\boldsymbol{H_d}$ obtained from micromagnetic simulations, against the applied field angle θ, as a thick red line in **Figure S5**. We also plot the simulated $\boldsymbol{H_d}$ from the macrospin model described in **S2.1** with a series of different demagnetising factors in different colours. Here, the demagnetising factors [$N_{x'}$ $N_{y'}$ $N_{z'}$] used correspond to the local axes in each section, and the demagnetising field is calculated in local coordinates and then converted into the global coordinates.

For Section 1 and Section 2 (**Figure S5 a, b**), we find that N = [0 0.4 0.6] (yellow line) matches the micromagnetic simulation the best. From the micromagnetic simulation, we observe that the component of the demagnetising field in the y direction is about three orders of magnitude smaller than components in the x and z direction and hence it is not considered

here. For section 3, we find N = [0.06 0.35 0.59] (orange line) fits best to the micromagnetic simulation as shown in **Figure S5c**. For section 3, a range of $N_{x'}$ from 0 to 0.1 were tested and $N_{x'}$ = 0.06, which gives the best results, is plotted here. After finding the demagnetising factor for each section, the simple macrospin model is used to determine the magnetisation $\boldsymbol{m}$ and demagnetising field $\boldsymbol{H_d}$ for external magnetic fields applied in different directions. These data are then used in the FEM analysis for the MR calculations.

S3 Magnetoresistance (MR) calculation using Finite Element Methods (FEM)

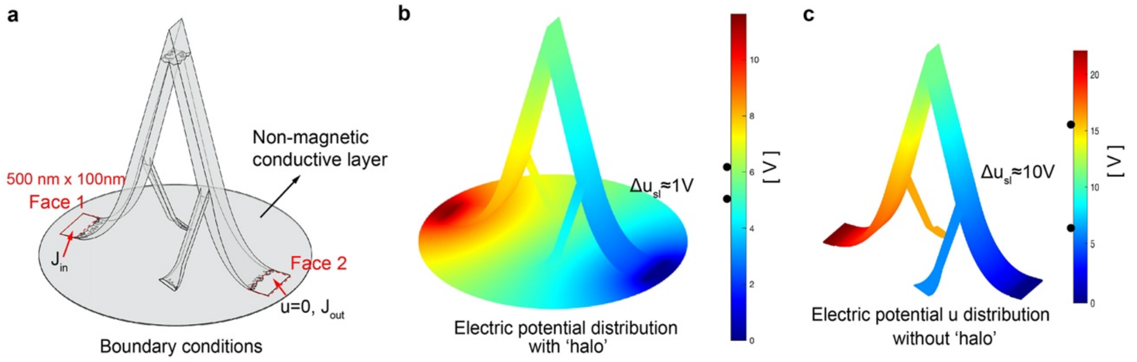

Figure S6 | FEM simulation. a, Boundary conditions. **b,** The simulated electric potential distribution with a conductive layer underneath the bridge. **c,** The simulated electric potential distribution for the bridge only.

S3.1 Boundary conditions

To calculate the MR signal, we assume a constant current supplied to the bridge and we solve the electric potential u in $\nabla \cdot [\sigma(\nabla u)] = 0$, using FEM simulations. The resistivity tensor (σ^{-1}) in the simulation is described in the main paper, and the boundary conditions are described here. First, we assume a constant current of 1 A flowing into the bridge through Face 1 and out of the bridge through Face 2, as shown in **Figure S6a**. Thus, the current flowing in and out of the nanobridge is set using Neumann boundary conditions as $\vec{n} \cdot (\sigma \nabla u) = -J_{in}\ or\ J_{out}$, respectively. Here $\vec{n}$ is the outward unit normal and $J_{in} = J_{out} = 1\text{A}/(500\ \text{nm} \times 100\text{nm})$ is the current density at Face 1 and 2. Secondly, we set the electric potential to be 0 at Face 2, as a Dirichlet boundary condition $u = 0$. Finally, for all other Faces in this model, we set $\vec{n} \cdot (\sigma \nabla u) = 0$, as no current flows in or out of the model through other Faces.

S3.2 Materials properties used and unintended deposition around the 3D nanobridge

The 3D nanobridge is deposited by FEBID with 30 kV acceleration voltage and 0.34 nA beam current using $Co_2(CO)_8$ as precursor. Similar growth conditions as the ones used here for 2D

deposits lead to a metallic nanocrystalline material, formed by cobalt crystals with typical sizes around 5-10 nm, and atomic percentages of ≈ 90-95% Co.[3,4] In our case, the larger beam currents in combination with a 3D geometry both enhance the local heating,[5] which is likely to promote higher Co purity *via* autocatalytic effects,[6–11] enhancing the electrical conduction properties of the material. Local heating during 3D growth is also likely to enhance the Co content, crystallinity, and magnetic properties.[12] Taking into account the lowest resistivity value reported so far for FEBID, equal to 26 μΩcm in reference,[6] and the typical resistivity of polycrystalline cobalt thin films deposited by conventional physical vapour deposition methods,[13–16] we thus expect the resistivity of the probed region of the nanobridge to be in the range of 11-26 μΩcm.[13–16]

We first substitute the resistivity ρ_{Co} =11 μΩcm into the bridge model shown in **Figure S6c**, which gives as a result a simulated voltage across the side contacts of about 10 V, which is 10 times larger than the resistance experimentally measured. Since the resistivity of FEBID Co is not likely to be smaller than the resistivity of polycrystalline cobalt thin films, this small measured resistance is attributed to an unintended deposition ('halo') around the desired 3D nanostructure. This 'halo' effect is caused by precursor dissociation by secondary and backscattered electrons reaching distances far beyond the primary electron beam, a common effect in FEBID.[17] This indicates the FIB milled trenches explained in the main manuscript did not completely prevent the influence of this 'halo'. Since this parasitic deposit is reported to have low cobalt concentration,[18–20] here it is modelled as a non-magnetic, round thin film as shown in **Figure S6a**. With this 'halo' included in the FEM model, the simulated resistance is reduced by approximately a factor of 10, and we reach a good quantitative agreement between experiments and simulations (**Figure S6b**).

The final magneto-transport parameters used in the simulation are: ρ_{Co} =15 μΩcm, AMR ratio = 0.95%[3] ρ_{AHE} = 0.56 μΩcm and $D(180K) = 390\ \mathrm{meV\mathring{A}}^2$.[13,21] Since the electron-magnon scattering model[21] developed by Raquet *et al.* only indicates the proportionality, but not the absolute magnitude of $\Delta\rho_{mmr}$, a factor of 15 is used to produce the best fit to the data.

To determine the reproducibility of our results, in addition to the sample used in the paper (**Figure S7a**), we also measured a smaller subset of data for a second structure with similar geometry as shown in **Figure S7b**. Specifically, we compare the θ = 0° and θ = 90° hysteresis

loops for both samples for a field range of -3 T to 3 T. The sample presented in the paper (**Figure S7c**) and the second sample (**Figure S7d**) exhibit similar resistance and a very similar trend of the data for the θ = 0° and θ = 90° cases, demonstrating that the realisation of the 3D nanomagnetic circuits are reproducible.

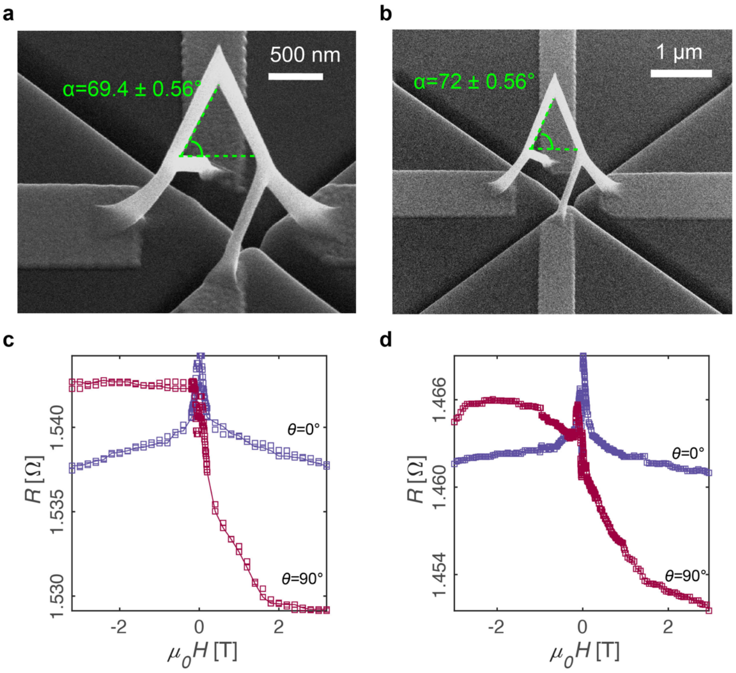

Figure S7 | a-b, The SEM images taken for the sample described in the paper and the supporting sample, respectively. **c-d**, The θ = 0° and θ = 90° measurements for the sample described in the paper and The supporting sample, respectively.

S3.3 Ordinary Hall effect and anomalous Hall effect

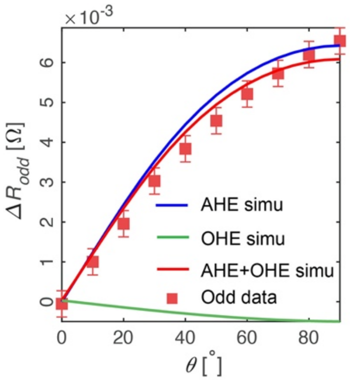

Figure S8 | The simulated results for AHE, OHE and their sum.

In the main paper, the sum of the ordinary and anomalous Hall effect is shown in **Figure 3b**. Here, we compare the contribution of each effect separately. The simulated results of AHE, OHE and their sum are plotted in **Figure S8**, where it is clear that AHE is the dominant effect. Values of $\rho_{\mathrm{AHE}} = 5.6 \times 10^{-9} \Omega\mathrm{m}$ and $R_{\mathrm{OHE}} = -1.2 \times 10^{-10}\ \Omega\mathrm{m/T}$ for the anomalous Hall

resistivity and the ordinary Hall coefficient, respectively, are used to produce the best fit to the data.

S4 Influence of heat

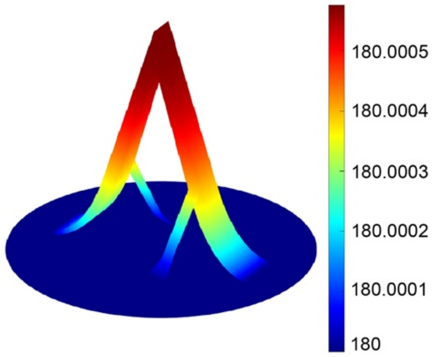

Figure S9| Temperature increase in the 3D nanobridge due to joule heating.

Temperature gradients are known to lead to electrical and spin transport effects.[22] Here, we simulate the temperature increase of the 3D nanobridge due to joule heating, as 3D nanostructures cannot dissipate heat to the substrate as well as 2D structures do. Here, we use the same 3D model as the one detailed above, finding the steady-state solution for the general heat equation,

$$\rho c_p \frac{\partial T}{\partial t} - \nabla \cdot (k \nabla T) = Q,$$

where ρ is the density, c_p is the specific heat capacity, k = 3 W/mK[23] is the heat conductivity, Q is the internal heat source. The first term can be ignored in our case, as we only evaluate the steady-state solution. The source of heat generation here is the joule heating and can be written as

$$Q = \sigma |\nabla V|^2.$$

In the experiment, a constant current of 0.6 μA is supplied, which is equivalent to a current density of 2.7×10^7 A/m^2. We simulate the voltage drop across the side contacts with this current density and then obtain the heat generation. The halo is set to have a constant temperature of 180 K, and convection though cool helium gas used in experiments, and radiation, are ignored. As shown in **Figure S9**, the temperature increase obtained for our 3D geometry is only 0.5 mK, which is negligible for our magnetotransport studies.

References

(1) Bertotti, G. Chapter 3 - Maxwell's Equations in Magnetic Media. In *Electromagnetism*; Academic Press: San Diego, 1998; pp 73–102.

(2) Abert, C.; Exl, L.; Bruckner, F.; Drews, A.; Suess, D. Magnum.Fe: A Micromagnetic Finite-Element Simulation Code Based on FEniCS. *J. Magn. Magn. Mate.r* **2013**, *345*, 29–35.

(3) Fernández-Pacheco, A.; Teresa, J. M. D.; Córdoba, R.; Ibarra, M. R. Magnetotransport Properties of High-Quality Cobalt Nanowires Grown by Focused-Electron-Beam-Induced Deposition. *J. Phys. D. Appl. Phys.* **2009**, *42*, 055005.

(4) Córdoba, R.; Lavrijsen, R.; Fernández-Pacheco, A.; Ibarra, M. R.; Schoenaker, F.; Ellis, T.; Barcones-Campo, B.; Kohlhepp, J. T.; Swagten, H. J. M.; Koopmans, B.; Mulders, J. J. L.; Teresa, J. M. D. Giant Anomalous Hall Effect in Fe-Based Microwires Grown by Focused-Electron-Beam-Induced Deposition. *Journal of Physics D.: Applied Physics* **2012**, *45*, 035001.

(5) Mutunga, E.; Winkler, R.; Sattelkow, J.; Rack, P. D.; Plank, H.; Fowlkes, J. D. Impact of Electron-Beam Heating during 3D Nanoprinting. *ACS Nano* **2019**, *13*, 5198–5213.

(6) Santos, M. V. P. dos; Velo, M.; Domingos, R. D.; Zhang, Y.; Maeder, X.; Guerra-Nunez, C.; Best, J. P.; Béron, F.; Pirota, K. R.; Moshkalev, S. A.; Diniz, J. A.; Utke, I. Annealing-Based Electrical Tuning of Cobalt-Carbon Deposits Grown by Focused-Electron-Beam-Induced Deposition. *ACS Appl. Mater. Inter.* **2016**, *8*, 32496-32503.

(7) Utke, I.; Hoffmann, P.; Melngailis, J. Gas-Assisted Focused Electron Beam and Ion Beam Processing and Fabrication. *J. Vac. Sci. Technology B Microelectron Nanometer Struct.* **2008**, *26*, 1197.

(8) Teresa, J. M. D.; Fernández-Pacheco, A.; Córdoba, R.; Serrano-Ramón, L.; Sangiao, S.; Ibarra, M. R. Review of Magnetic Nanostructures Grown by Focused Electron Beam Induced Deposition (FEBID). *J. Phys. D. Appl. Phys.* **2016**, *49*, 243003.

(9) Belova, L. M.; Dahlberg, E. D.; Riazanova, A.; Mulders, J. J. L.; Christophersen, C.; Eckert, J. Rapid Electron Beam Assisted Patterning of Pure Cobalt at Elevated Temperatures *via* Seeded Growth. *Nanotechnology* **2011**, *22*, 145305.

(10) Pablo-Navarro, J.; Sanz-Hernández, D.; Magén, C.; Fernández-Pacheco, A.; Teresa, J. M. de. Tuning Shape, Composition and Magnetization of 3D Cobalt Nanowires Grown by Focused Electron Beam Induced Deposition (FEBID). *J. Phys. D. Appl. Phys.* **2017**, *50*, 18LT01.

(11) Fernández-Pacheco, A.; Skoric, L.; Teresa, J. M. D.; Pablo-Navarro, J.; Huth, M.; Dobrovolskiy, O. V. Writing 3D Nanomagnets Using Focused Electron Beams. *Materials* **2020**, *13*, 3774.

(12) Pablo-Navarro, J.; Magén, C.; Teresa, J. M. de. Purified and Crystalline Three-Dimensional Electron-Beam-Induced Deposits: The Successful Case of Cobalt for High-Performance Magnetic Nanowires. *ACS Appl. Nano Mater.* **2017**, *1*, 38–46.

(13) Gil, W.; Görlitz, D.; Horisberger, M.; Kötzler, J. Magnetoresistance Anisotropy of Polycrystalline Cobalt Films: Geometrical-Size and Domain Effects. *Phys. Rev. B* **2005**, *72*, 134401.

(14) Gurp, G. J. van. Resistivity, Grain Size, and Structure of Vacuum-Deposited Co Films. *J. Appl. Phys.* **1975**, *46*, 1922–1927.

(15) Pal, A. K.; Chaudhuri, S.; Barua, A. K. The Electrical Resistivity and Temperature Coefficient of Resistivity of Cobalt Films. *J. Phys. D. Appl. Phys.* **1976**, *9*, 2261–2267.

(16) Vries, J. W. C. D. Temperature and Thickness Dependence of the Resistivity of Thin Polycrystalline Aluminium, Cobalt, Nickel, Palladium, Silver and Gold Films. *Thin Solid Films* **1988**, *167*, 25–32.

(17) Plank, H.; Smith, D. A.; Haber, T.; Rack, P. D.; Hofer, F. Fundamental Proximity Effects in Focused Electron Beam Induced Deposition. *ACS Nano* **2011**, *6*, 286–294.

(18) Nikulina, E.; Idigoras, O.; Porro, J. M.; Vavassori, P.; Chuvilin, A.; Berger, A. Origin and Control of Magnetic Exchange Coupling in between Focused Electron Beam Deposited Cobalt Nanostructures. *Appl. Phys. Lett.* **2013**, *103*, 123112.

(19) Lau, Y. M.; Chee, P. C.; Thong, J. T. L.; Ng, V. Properties and Applications of Cobalt-Based Material Produced by Electron-Beam-Induced Deposition. *J. Vac. Sci. Technology Vac. Surfaces Films* **2002**, *20*, 1295–1302.

(20) Boero, G.; Utke, I.; Bret, T.; Quack, N.; Todorova, M.; Mouaziz, S.; Kejik, P.; Brugger, J.; Popovic, R. S.; Hoffmann, P. Submicrometer Hall Devices Fabricated by Focused Electron-Beam-Induced Deposition. *Appl. Phys. Lett.* **2005**, *86*, 042503.

(21) Raquet, B.; Viret, M.; Sondergard, E.; Cespedes, O.; Mamy, R. Electron-Magnon Scattering and Magnetic Resistivity in 3D Ferromagnets. *Phys. Rev. B* **2002**, *66*, 024433.

(22) Mizuguchi, M.; Nakatsuji, S. Energy-Harvesting Materials Based on the Anomalous Nernst Effect. *Sci. Technol. Adv. Mat.* **2019**, *20*, 262–275.

(23) Pradhan, N. R. Thermal Conductivity of Nanowires, Nanotubes and Polymer-Nanotube Composites. Ph.D., Worcester Polytechnic Institute, Worcester, April 2010.
